# Diet patterns and cognitive performance in a UK Female Twin Registry (TwinsUK)

**DOI:** 10.1186/s13195-024-01387-x

**Published:** 2024-01-23

**Authors:** Claire T. McEvoy, Amy Jennings, Claire J. Steves, Alexander Macgregor, Tim Spector, Aedin Cassidy

**Affiliations:** 1 The Institute for Global Food Security, Queen’s University Belfast, Belfast, Northern Ireland UK; 2 The Global Brain Health Institute, University of California San Francisco, San Francisco, CA USA; 3 The Global Brain Health Institute, Trinity College Dublin, Dublin, Ireland; 4 Department of Twin Research & Genetic Epidemiology, King’s College London, St Thomas’ Campus, London, UK; 5 Norwich Medical School, University of East Anglia, Norwich, UK; 6 Centre for Public Health, Institute of Clinical Sciences B, School of Medicine, Dentistry and Biomedical Sciences, Queen’s University Belfast, Belfast, Northern Ireland BT12 6BJ UK

**Keywords:** Twins, Dietary patterns, Cognitive performance, Gut microbiome

## Abstract

**Background:**

Plant-based diets may provide protection against cognitive decline and Alzheimer’s disease, but observational data have not been consistent. Previous studies include early life confounding from socioeconomic conditions and genetics that are known to influence both cognitive performance and diet behaviour. This study investigated associations between Mediterranean (MED) diet and MIND diets and cognitive performance accounting for shared genotype and early-life environmental exposures in female twins.

**Methods:**

Diet scores were examined in 509 female twins enrolled in TwinsUK study. The Cambridge Neuropsychological Test Automated Battery was used to assess cognition at baseline and 10 years later (in *n* = 275). A co-twin case–control study for discordant monozygotic (MZ) twins examined effects of diet on cognitive performance independent of genetic factors. Differences in relative abundance of taxa at 10-year follow-up were explored in subsamples.

**Results:**

Each 1-point increase in MIND or MED diet score was associated with 1.75 (95% *CI*: − 2.96, − 0.54, *p* = 0.005 and *q* = 0.11) and 1.67 (95% *CI*: − 2.71, − 0.65, *p* = 0.002 and *q* = 0.02) fewer respective errors in paired-associates learning. Within each MZ pair, the twin with the high diet score had better preservation in spatial span especially for MED diet (*p* = 0.02). There were no differences between diet scores and 10-year change in the other cognitive tests. MIND diet adherence was associated with higher relative abundance of Ruminococcaceae UCG-010 (0.30% (95% *CI* 0.17, 0.62), *q* = 0.05) which was also associated with less decline in global cognition over 10 years (0.22 (95% *CI* 0.06, 0.39), *p* = 0.01).

**Conclusions:**

MIND or MED diets could help to preserve some cognitive abilities in midlife, particularly episodic and visuospatial working memory. Effects may be mediated by high dietary fibre content and increased abundance of short-chain fatty acid producing gut bacteria. Longer follow-up with repeated measures of cognition will determine whether diet can influence changes in cognition occurring in older age.

**Supplementary Information:**

The online version contains supplementary material available at 10.1186/s13195-024-01387-x.

## Background

Loss of cognitive function during ageing is a public health concern due to its adverse impact on morbidity and mortality [1, 2]. Accelerated cognitive decline beyond that of normal ageing may also predict future Alzheimer’s disease (AD) [3]. While decline in some cognitive abilities has been shown from midlife [4], there is considerable interindividual variability in the rate of cognitive decline which is influenced by cardiovascular risk factors and lifestyle behaviours [5]. Identifying effective strategies to optimize cognitive function is critical for AD risk reduction [2]. In this regard, growing evidence supports nutrient-dense dietary patterns, particularly the Mediterranean (MED) and the Mediterranean-dietary approaches to stop hypertension intervention for neurodegenerative delay (MIND) diets for neuroprotection [6]. Furthermore, data suggest that beneficial effects of MED diet on cognitive performance are mediated by positive changes to the gut microbiome [7].

The MED diet is rich in fruit and vegetables, whole grains and legumes, moderate in fish and nuts and alcohol and generally low in red meats [8] and proven to be cardioprotective [9]. Similarly, the MIND diet [10] is also a plant-rich dietary pattern but differs from the MED by a greater emphasis on berries and green leafy vegetables that are independently linked to brain health. Greater adherence to MIND and MED diets has been associated with slower cognitive decline [11], preservation of brain structures [12], and reduced AD risk in later life [6, 10, 13]. However, results have been inconsistent [6] partly due to the variation in measurement of cognitive outcomes. Methodological challenges also limit the inference of causal associations as studies have been conducted primarily in older populations, often with short follow-up time and limited information on earlier life dietary exposure. This means they are particularly susceptible to reverse causation bias because older adults may already have advanced age-related brain changes or AD pathology that can alter their dietary behaviour [14]. Further longitudinal studies initiated in younger cognitively healthy populations are required to determine the strength of association between MIND and MED diets and subsequent cognitive decline.

Previous studies have also been unable to account for early life confounding from parental lifestyle, socioeconomic conditions and genetics that are known to influence both cognitive performance and dietary behaviour [15]. Studying monozygotic (MZ) twins who are genetically identical, and dizygotic (DZ) twins who share around half their genes, offers a unique opportunity to quantify the effect of diet on cognitive function after controlling for shared genotype and early-life environmental and nutritional exposures. To our knowledge, the impact of shared familial factors on relations between neuroprotective diets, gut microbiota and cognitive performance has not yet been investigated.

We studied a cohort of cognitively healthy female twins to (1) determine the cross-sectional relationships between MED and MIND diet scores and cognitive function, (2) determine longitudinal relations between the diet scores and 10-year change in cognitive performance, and (3) quantify the influence of MED and MIND diets on cognitive performance independent of genetic and other environmental factors in discordant MZ twin pairs. Furthermore, in a subsample of female twins, we explored gut microbiome as a potential mechanistic pathway between diet and cognitive performance.

## Methods

### Population

**Fig. 1:**
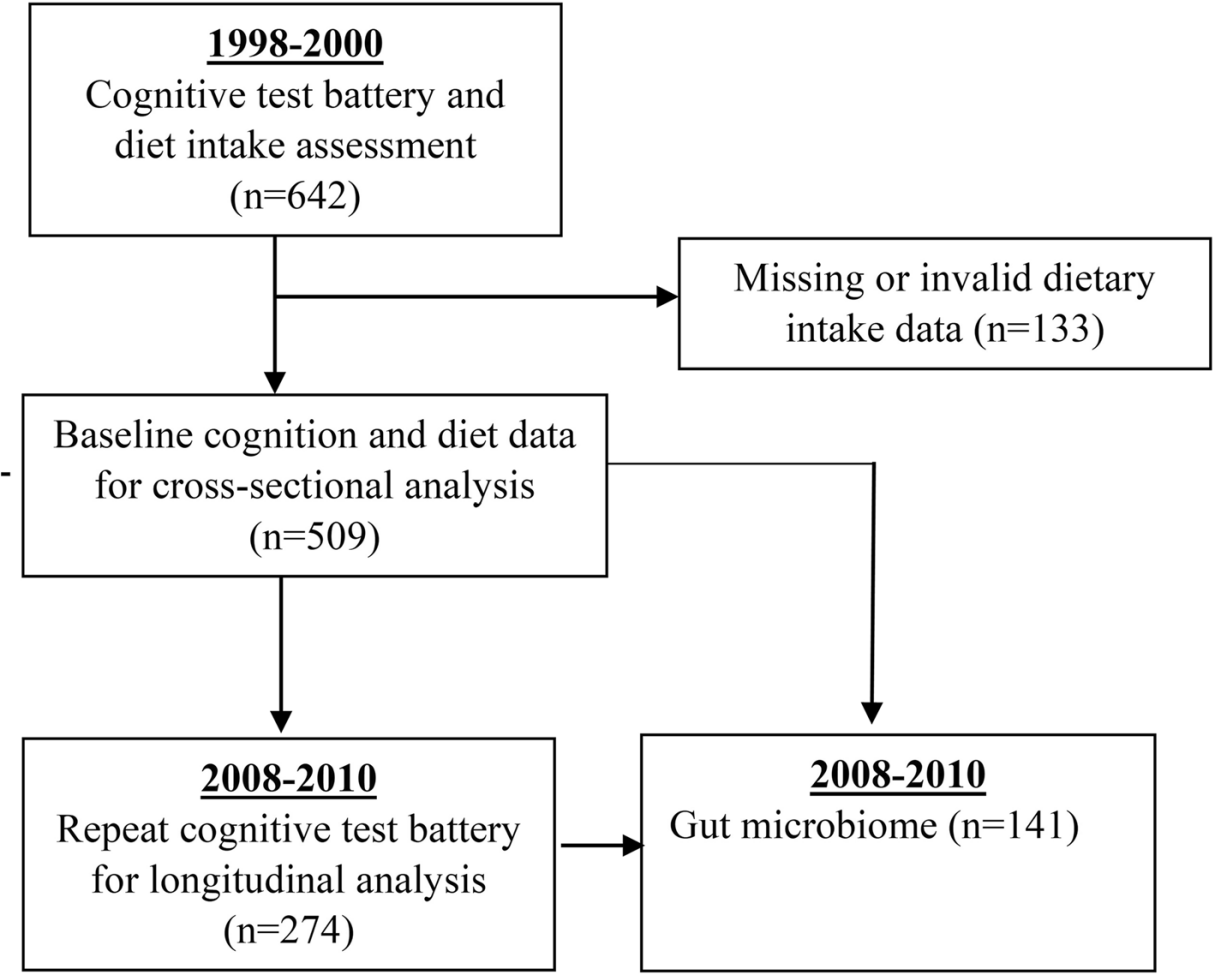
Summary of participants in the study

Data for this study were drawn from the UK Adult Twin Registry (TwinsUK) that enrolled healthy volunteers from media campaigns in the UK and Ireland between 1992 and 2004 [16]. The primary aim of TwinsUK was to investigate diseases affecting women during ageing; therefore, the initial recruitment was targeted towards female twins. Ethical approvals for the study were granted from St. Thomas’ Hospital Research Ethics Committee, and informed consent was obtained from all participants. For the current study, we included participants who had completed both the baseline diet and cognitive assessments (*n* = 642) between 1998 and 2000. After excluding those with implausible or missing dietary intake data (*n* = 133), the final analytic sample comprised 509 female twins (21 individuals and 244 twin pairs) as shown in Fig. 1. Of these, 34% were MZ, and 66% were DZ twins as assessed by the ‘peas in a pod’ questionnaire [17] and confirmed where necessary by multiplex DNA fingerprinting (PE, Applied Biosystems). There were no triplets in the analytic sample. The cognitive assessment was repeated after 10 years (2008–2010) in up to 274 of the sample. Those who did not complete the repeat cognitive assessment were more likely to be younger with greater educational attainment at baseline (all *p* < 0.01).

### Diet assessment

Baseline dietary intake was assessed using a 131-item semiquantitative food frequency questionnaire (FFQ) [18] previously validated for the UK population [19]. From the FFQ data, we calculated two diet scores: an a priori MIND diet score [10] and a population-specific MED diet score [8] as outlined below.

#### MIND diet score

Food items from the FFQ were aggregated into 15 dietary components in accordance with predefined MIND diet score criteria [10]. Ten components were considered brain healthy food groups (green leafy vegetables, other vegetables, nuts, berries, beans, whole grains, seafood, poultry, olive oil, and wine), and five were considered brain unhealthy food groups (red meats, butter and stick margarine, cheese, pastries and sweets and fried/fast food). Each dietary component was assigned a score of 0, 0.5 or 1 depending on the reported intake. Scores for the healthy components increased monotonically with higher consumption, and reverse scores were used for the five unhealthy components. Information for olive oil was not available so the ratio of monounsaturated to saturated fatty acid (MUFA: SFA) intake was used and scored 0, 0.5 or 1 point, with a ratio ≥ 2 assigned the highest score [20]. Wine (red and/or white wine) was awarded 0 points if no wine consumed or intake was > 1 glass/day, 1 point for 1 glass wine/day and 0.5 for other wine intake up to 6 glasses/week. Dietary component scores were summed to obtain an overall score ranging from 0 to 15, where higher scores indicate greater adherence to the MIND diet.

#### MED diet score

The MED diet score was based on the population median intakes of nine dietary components: fruits and nuts, vegetables, legumes, dairy products, cereals, meat and meat products, fish, alcohol and the MUFA: SFA [8]. Healthy food components (fruits and nuts, vegetables, legumes, cereals, fish and MUFA/SFA were scored 1 point if consumption was above the population median and scored 0 point if intake was below the population median. The score system was reversed for population median intakes of high-fat dairy products, meat and meat products. Women who consumed 5–25 g alcohol/day were awarded 1 point, while those outside this range were scored 0 point. The total score ranged from 0 to 9 points, where higher scores indicate greater concordance with the MED dietary pattern.

### Cognitive outcomes

Cognitive function was assessed by the Cambridge Automated Neuropsychological Test Battery (CANTAB) performed at baseline and repeated after 10 years. CANTAB is an automated series of short tests designed to evaluate cognitive function and completed by the participant on a touch screen computer as previously described [21]. In this study, six cognitive tests were examined: (1) simple reaction time (SRT) to assess the speed of reaction, (2) spatial working memory (SWM) to assess the ability to retain and manipulate visuospatial information, (3) paired-associates learning (PAL) to assess episodic memory, (4) pattern recognition memory (PRM) to assess visual episodic memory, (5) delayed matching to sample (DMS) to assess decision time, and (6) spatial span (SSP) to assess visuospatial working memory capacity.

We examined the 10-year change in the individual cognitive test scores and an age-related global cognition score (ARC) previously derived from principal components analysis using 10-year change in individual cognitive test scores [21]. The ARC factor explained 25% of the variance in the female twins and was strongly associated with age (standardized beta − 0.066 *p* < 0.001) [21]. A more positive ARC score indicated less decline in global cognition after 10 years.

### Gut microbiome

At the 10-year follow-up, a subsample of participants provided a faecal sample for gut microbiome analysis as described previously [22]. In brief, samples were stored at − 80 °C before being batch shipped to Cornell University (Ithaca, USA) for 16S rRNA gene sequencing. In the current study, we explored the relative abundance of genus level microbial taxa.

### Baseline covariates

Self-reported covariates included the following: age (years), educational attainment based on the UK education system (no qualifications; O-level, GCSE, or equivalent; Scottish higher, A level, or equivalent; university degree or postgraduate degree), smoker (yes; no), postmenopausal status, diabetes (self-reported diagnosis and/or antidiabetic medication use), cardiovascular disease (myocardial infarction and/or angina) and physical activity level (inactive, moderately active, active). The country-specific multiple deprivation index (IMD) was the measure of socioeconomic status [21] where quintile 1 = highest deprivation and quintile 5 = least deprivation. Clinic measured covariates were body mass index (BMI, kg/m^2^) calculated from measured weight and height and hypertension (based on measured systolic ≥ 140 mmHg and/or diastolic ≥ 90 mmHg).

### Statistical analysis

We analysed MIND and MED diet scores as both a continuous variable (per 1-point increase) and a categorical variable of low and high adherence score based on a median split for each score. Participant characteristics were compared across categories of diet scores using descriptive statistical tests. Two sample *t*-test was used for continuous variables, and chi-square test was used for categorical variable. A multivariable general linear model was applied to examine associations between the diet scores and performance on the individual cognitive test scores. Cognitive test scores were transformed for cross-sectional analysis as described for the TwinsUK cohort [21]. Separate models were estimated for diet scores as continuous variables and as categories of high and low adherence with low adherence as the reference category. Models were adjusted first for the effects of baseline demographics (age, SES and educational attainment) and then for health and lifestyle factors (energy intake, BMI, physical activity, postmenopausal status, current smoking and hypertension).

Next, we applied multivariate linear models to examine MIND and MED diet scores in relation to 10-year change in cognitive performance. For the longitudinal analysis, we first adjusted the models for interindividual differences in baseline cognitive function where applicable, and then subsequent models were adjusted for covariates [23] using the same approach described above. Sensitivity analyses were carried out to adjust models for multivitamin supplement use.

In the co-twin case–control study, paired *t*-tests were used to estimate the difference in neurocognitive outcomes in discordant MZ twin pairs defined as > 1 point difference for each diet score. Two-sided *p*-values < 0.05 were considered statistically significant for the co-twin analyses.

For the gut microbiome exploratory analyses, we compared the relative abundance of taxa at 10-year follow-up between participants with low and high diet scores at baseline. For the taxa where significant associations were observed, we examined if relative abundance was associated with 10-year change in cognitive performance. To reduce random error in low abundance taxa, we focused our analysis on the core measurable microbiota, which in this dataset excluded taxa if relative abundance was below 0.01% in at least 10% of samples, leaving 135 taxa at the genus level. A multiple testing correction was applied to linear models and microbial taxa abundances using the Benjamini–Hochberg method for false discovery rate where a *q*-value < 0.20 was considered statistically significant. Analyses were performed using StataCorp. 2017 (Stata Statistical Software: Release 15. (Version 15.1), College Station, TX, USA: StataCorp LLC).

## Results

At baseline, women were 51.9 ± 12.5 years (range 18–79 years). Mean MIND diet score was 7.6 ± 1.49 (range 3–11.5), and mean MED diet score was 4.42 ± 1.74 (range 0–9) indicating moderate adherence to the dietary patterns. The diet scores were positively correlated (*r* = 0.50, *p* < 0.001) in the sample. As shown in Table 1, women with high MIND diet scores were older with lower BMI and more likely to be hypertensive and consume fewer vitamin supplements, compared to those with low MIND diet score. There were few differences between the women with high and low adherence to MED diet, although those with high MED diet score were less likely to smoke compared to those with low diet score.

**Table 1:** Characteristics of female twins by low and high categories of MIND and MED diet scores (*n* = 509)

	**MIND diet score (0–15)**			**MED diet score (0–9)**		
	**Low (0.0–7.5)**	**High (8.0–15)**	***p*-*value***	**Low (0–4)**	**High (5–9)**	***p*-*value***
**Mean (SD) or *n* (%)**	***n* = 285**	***n* = 224**		***n* = 280**	***n* = 229**	
**Age, years**	50.9 (13.3)	53.2 (11.4)	0.04	51.2 (13.0)	52.8 (11.8)	0.15
**Monozygotic twin**	104 (36)	69 (31)	0.18	91 (33)	82 (36)	0.43
**Education, no qualifications**	26 (9)	27 (12)	0.61	25 (9)	28 (12)	0.57
**Lower socioeconomic status**^**a**^	45 (16)	36 (16)	0.93	44 (16)	37 (16)	0.89
**Current smoker**	47 (16)	29 (13)	0.27	51 (18)	25 (11)	0.02
**Body mass index, kg/m**^**2**^	25.9 (4.7)	24.7 (3.7)	0.001	25.5 (4.5)	25.2 (4.1)	0.51
**Physically inactive**	82 (29)	47 (21)	0.10	80 (29)	49 (21)	0.12
**Postmenopausal**	156 (55)	140 (63)	0.08	166 (59)	130 (57)	0.58
**Hypertension**	62 (22)	68 (30)	0.03	75 (27)	55 (24)	0.48
**Diabetes**^**b**^	5 (3)	6 (4)	0.69	6 (4)	5 (3)	0.78
**Cardiovascular disease**^**c**^	6 (4)	7 (5)	0.69	3 (2)	10 (6)	0.05
**Calories, Kcal/day**	2092 (549)	1925 (504)	< 0.001	1955 (520)	2095 (544)	0.003
**Dietary fibre, g/day**	19.7 (6.9)	22.9 (7.1)	< 0.001	17.8 (6.2)	24.5 (6.5)	< 0.001
**Vitamin supplement**	147 (55)	148 (68)	0.003	155 (58)	140 (64)	0.18
**Diet score**	6.5 (0.93)	8.9 (0.85)	< 0.001	3.11 (1.00)	6.02 (0.96)	< 0.001

^a^Quintiles 1 and 2 of the country-specific multiple deprivation index were considered as lower socioeconomic status

^b^Available in 315 participants

^c^Available in 314 participants

### Diet patterns and baseline cognitive performance

There was no association between MED diet score and baseline cognitive test scores (Additional file 1: Table S1). After adjustment for demographic, health and lifestyle covariates, each 1-point increase in MIND diet score was associated with faster reaction time (SRT) and better visual episodic memory (PRM) (both *p* < 0.05) but was nonsignificant after correcting for multiple comparison (*q* = 0.35). SWM, PAL, DMS and SSP test scores did not significantly differ by increasing MIND diet score (Additional file 1: Table S1).

### Diet patterns and 10-year change in cognitive performance

Table 2 shows the association (β (95% CI)) of 1-point increase in MIND and MED diet scores with 10-year change in cognitive test scores. Women with higher MIND tended to have better ARC; however, increasing MIND and MED scores was not related to overall ARC score in multivariate models. For the individual cognitive scores, increasing adherence to either MIND or MED diet was associated with fewer errors on the PAL test. After adjustment for demographics, lifestyle and health factors, each 1-point increase in MIND or MED score was associated with 1.75 (95% *CI*: − 2.96, − 0.54, *p* = 0.005; *q* = 0.11) and 1.67 (95% *CI*: − 2.71, − 0.65, *p* = 0.002; *q* = 0.02) fewer PAL errors respectively. Adjusting the models further for vitamin supplement use did not substantially alter the results for MED diet but slightly attenuated the estimates for MIND diet score [1.60 (95% *CI*: − 2.87, − 0.33]. The findings were similar in categorical models of low and high diet scores. In multivariable adjusted models, women with high adherence to MIND and MED diets had better preservation of PAL performance after 10 years compared to women with low adherence (*p* < 0.05; *q* = 0.21) (Additional file 1: Table S2). High versus low diet scores were not related to 10-year change in the other cognitive tests.

**Table 2:** 10-year change in cognitive performance by increasing diet score in the female twins (*n* = 275 max)

		**Per 1-point increase in MIND diet score**			**Per 1-point increase in MED diet score**		
**Cognitive scores**		**β (95% *CI*)**			**β (95% *CI*)**		
	***n***	Basic	Model 1	Model 2	Basic	Model 1	Model 2
**Age-related cognition**	220	− 0.01 (− 0.10, 0.08)	− 0.03 (− 0.12, 0.05)	0.02 (− 0.11, 0.06)	− 0.01 (− 0.07, 0.09)	− 0.01 (− 0.08, 0.06)	− 0.02 (− 0.09, 0.05)
**Change in SRT (ms)**	254	0.51 (− 3.80, 4.82)	0.85 (− 3.38, 5.07)	1.13 (− 3.32, 5.59)	− 0.78 (− 4.65, 3.09)	− 0.66 (− 4.47, 3.15)	− 0.39 (− 4.24, 3.46)
**Change in SWM (total errors)**	274	− 0.29 (− 1.44, 0.87)	− 0.08 (− 1.21, 1.05)	− 0.34 (− 1.49, 0.81)	− 0.55 (− 1.57, 0.47)	− 0.47 (− 1.47, 0.54)	− 0.43 (− 1.41, 0.55)
**Change in PAL (total errors)**	275	− 1.53* (− 2.70, − 0.35)	− 1.41** (− 2.58, − 0.24)	− 1.75* (− 2.96, − 0.54)	− 1.76** (− 2.79, − 0.73)	− 1.66** (− 2.69, − 0.63)	− 1.67** (− 2.71, − 0.65)
**Change in PRM (total correct)**	274	− 0.08 (− 0.25, 0.09)	− 0.09 (− 0.26, 0.08)	− 0.05 (− 0.22, 0.13)	− 0.07 (− 0.22, 0.08)	− 0.08 (− 0.23, 0.07)	− 0.09 (− 0.24, 0.06)
**Change in DMS (total correct)**	274	0.17 (− 0.08, 0.42)	0.11 (− 0.13, 0.35)	0.15 (− 0.10, 0.39)	0.18 (− 0.04, 0.40)	0.13 (− 0.08, 0.34)	0.14 (− 0.07, 0.35)
**Change in SSP (span)**	274	0.03 (− 0.05, 0.10)	0.02 (− 0.06, 0.09)	0.03 (− 0.04, 0.11)	0.02 (− 0.05, 0.08)	0.02 (− 0.05, 0.08)	0.01 (− 0.05, 0.08)

Basic adjusted for baseline cognitive test score except for age-related cognition score. Model 1 adjusted for age, SES, educational attainment; model 2, model 1 + energy intake, BMI, physical activity, postmenopausal status, current smoking, and hypertension. *p < 0.05 and *q* < 0.11; **p < 0.01 and *q* = 0.02

### Diet patterns and 10-year change in cognitive performance in the discordant MZ twin pairs

**Fig. 2:**
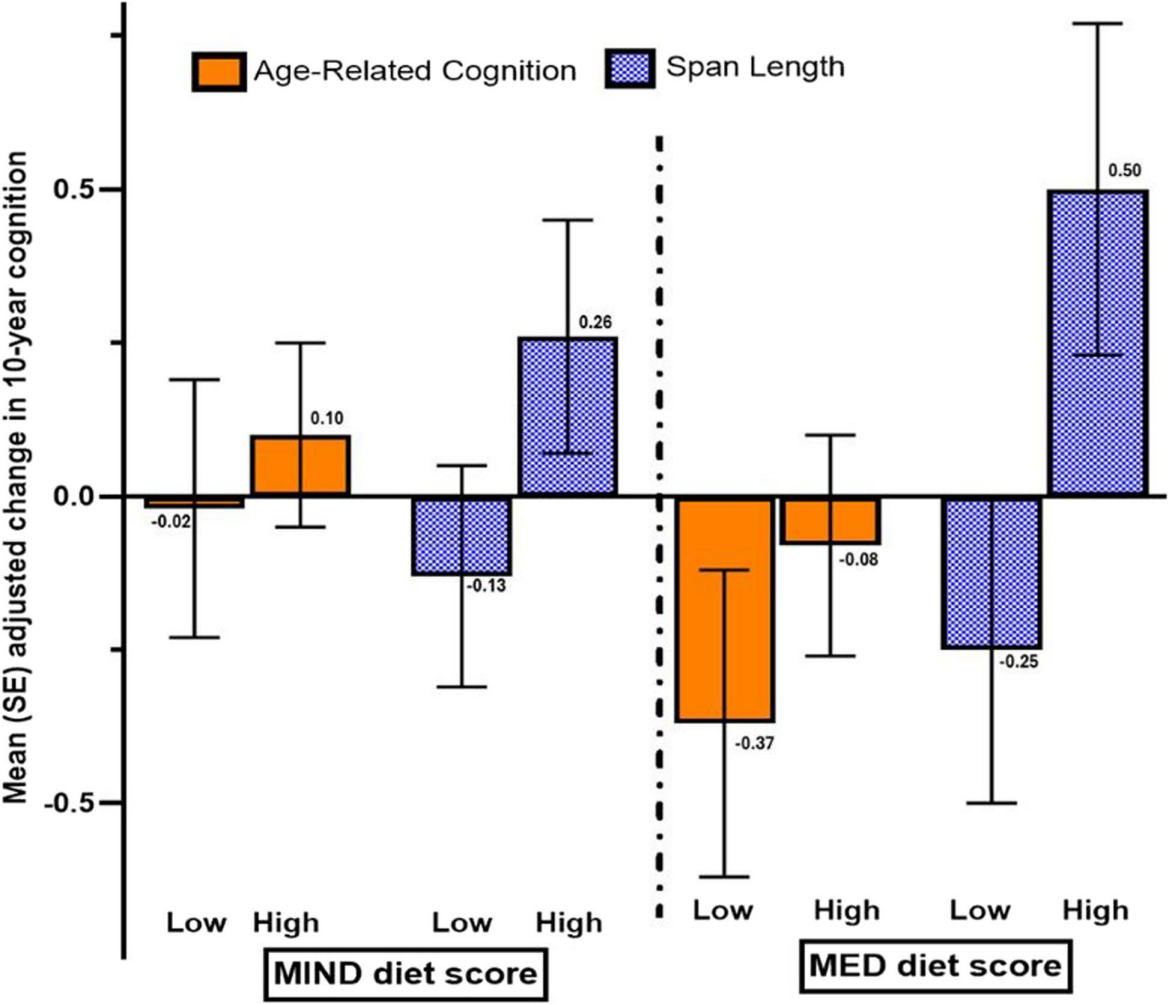
Change in adjusted mean (SE). Age-related cognition and spatial span length over 10 years in MZ twins discordant for MIND and MED diet score

For twins with 10-year cognitive change, 23 MZ pairs were discordant for MIND diet score, and 24 MZ pairs were discordant for MED diet score (≥ 1 point difference). There were no significant differences between discordant MZ twin pairs in demographics (SES, education), health (menopausal status, hypertension) or lifestyle behaviours (BMI, physical activity, smoking, calorie intake or multivitamin use). The mean difference in diet scores for discordant MZ pairs was 1.92 points for MIND (6.8 ± 1.20 versus 8.6 ± 1.30) and 2.62 points for MED (3.5 ± 1.35 versus 6.1 ± 1.41) (all *p* < 0.0001). In general, the MZ twin with high diet score had less decline in global cognition as measured using ARC over 10 years, but the difference was not significant as shown in Fig. 2. Within each MZ pair, the twin with the high MIND or MED diet score had greater preservation in spatial span length which was significant for the MED diet (*p* = 0.02, *q* = 0.11) (high versus low MIND *p* = 0.13). There were no significant differences between diet scores and 10-year change in the other cognitive tests for discordant MZ twins.

### Diet pattern and gut microbiome

High adherence to the MIND diet at baseline was associated with higher relative abundance of Ruminococcaceae UCG-010 (0.30% (95% *CI* 0.17, 0.62), *q* = 0.05) and lower relative abundance of *Dorea* (− 0.6 1% (95% *CI* − 1.0, − 0.4), *q* = 0.01) (Additional file 1: Table S3) at 10-year follow-up, compared to low adherence. These associations were nonsignificant when additionally adjusted for fibre intake. We observed no associations between adherence to the MED diet score and relative abundance of taxa (Additional file 1: Table S4).

Higher relative abundance of Ruminococcaceae UCG-010 was associated with less decline in global cognition on the ARC over 10 years (0.22 (95% *CI* 0.06, 0.39), *p* = 0.01; *q* = 0.16, Table 3) and improved 10-year change in SWM scores (− 3.5 total errors (95% *CI* − 6.43, − 0.57), *p* = 0.02; *q* = 0.21).

**Table 3:** 10-year change in cognitive scores and relative abundance of taxa at follow-up in female twins (*n* = 131)

**Cognitive scores**	**Taxa**	***n* = **	**β (95% *CI*)**	***q* = **
Age-related cognition	*Dorea*	109	− 0.02 (− 0.18, 0.13)	0.75
	Ruminococcaceae UCG-010	109	0.22 (0.06, 0.39)	0.16
	*Oxalobacter*	109	− 2.26 (− 7.22, 2.69)	0.50
Change in SRT (ms)	*Dorea*	123	11.19 (− 0.50, 22.89)	0.31
	Ruminococcaceae UCG-010	123	− 7.47 (− 21.83, 6.88)	0.50
	*Oxalobacter*	123	− 255 (− 575, 64.0)	0.37
Change in SWM (total errors)	*Dorea*	131	1.20 (− 1.41, 3.81)	0.50
	Ruminococcaceae UCG-010	131	− 3.50 (− 6.43, − 0.57)	0.21
	*Oxalobacter*	131	− 13.1 (− 78.8, 52.6)	0.73
Change in PAL (total errors)	*Dorea*	131	1.94 (− 0.76, 4.63)	0.37
	Ruminococcaceae UCG-010	131	− 0.91 (− 3.44, 1.62)	0.59
	*Oxalobacter*	131	17.8 (− 22.6, 58.3)	0.50
Change in PRM (total correct)	*Dorea*	131	0.24 (− 0.10, 0.58)	0.37
	Ruminococcaceae UCG-010	131	− 0.45 (− 0.86, − 0.03)	0.25
	*Oxalobacter*	131	− 2.92 (− 13.3, 7.5)	0.64
Change in DMS (total correct)	*Dorea*	131	− 0.28 (− 0.77, 0.22)	0.50
	Ruminococcaceae UCG-010	131	0.39 (− 0.18, 0.97)	0.37
	*Oxalobacter*	131	6.39 (− 6.9, 19.7)	0.50
Change in SSP (span)	*Dorea*	131	− 0.21 (− 0.45, 0.02)	0.31
	Ruminococcaceae UCG-010	131	0.19 (− 0.06, 0.45)	0.37
	*Oxalobacter*	131	1.89 (− 4.70, 8.49)	0.64

Values are beta coefficients (95% CI). Model adjusted for age, SES, educational attainment, energy intake, BMI, physical activity, postmenopausal status, current smoking, hypertension. *q*-value, false discovery rate adjusted *p*-value

## Discussion

To our knowledge, this is the first study investigating relations between neuroprotective dietary patterns and cognitive ageing in middle-aged female twins. Uniquely, we were also able to explore the potential mediating effect of gut microbiota on diet and cognition in the sample. The main finding was that higher MIND and MED diet scores were associated with better cognitive performance over 10 years in tests for episodic memory and visuospatial working memory. In the entire sample, women with higher adherence to either diet showed modest improvement in episodic memory performance on the PAL after adjustment for demographic, lifestyle, and health covariates. While improvement in cognitive scores can be attributed to artefactual gain or ‘practice effects’ from the initial test performance [24], this is unlikely to explain the current results given the long 10-year interval between testing. The same pattern for PAL performance was not replicated in the MZ co-twins; therefore, the possibility of residual confounding by unidentified genetic factors in the full sample cannot be excluded. In co-twin control study, twins with better adherence to the MIND, and particularly the MED diet, had less 10-year change in a visuospatial memory test. These results are important given that compromised episodic and working memory capacity has been implicated as early contributors to other cognitive deficits during ageing including long-term memory, problem-solving, decision-making and language that can impair the ability to perform everyday activities [5]. Overall, this study suggests that MIND or MED diets could help to preserve some cognitive abilities during in midlife. A future longitudinal follow-up of the twins with repeated measures of cognition will elucidate the links between diet and cognitive changes occurring in older age.

In contrast with studies involving older adults (aged ≥ 65 years) reporting benefits of MED and MIND on global cognition [11, 25, 26], the associations were not as strong in the female twins. It is possible that changes in cognitive function are of greater magnitude and more easily detected with cognitive tests in older than younger cognitively healthy adults. Only a small number of studies have involved younger populations, and findings are conflicting [23, 27–29]. Beneficial impacts of MED diet on midlife global cognitive performance have been demonstrated in adults with type 2 diabetes [27] and in a biracial population [23]; however, adherence to a healthy (prudent) dietary pattern at midlife was not associated with subsequent 18–20 years decline in global cognition [28, 29]. Discrepancies in results could be due to differences in score systems to derive dietary patterns as well as variation in cognitive test batteries, length of follow-up, and in adjustment for potential confounders. The present study was conducted in twins with the unique advantage of controlling for genetic and early nutritional and lifestyle factors that contribute to cognitive ageing and are difficult to measure in other population studies. Although observational data have been inconsistent, our findings lend support to accumulating experimental data showing small but beneficial effects of MED diet on individual cognitive domains including working memory, episodic memory, and visuospatial and executive function in cognitively healthy populations [30].

The mechanisms underpinning how MIND and MED diets influence cognitive ageing are not clear but are thought to include anti-inflammatory, metabolic and neurovascular pathways [31].

Causal links between gut dysbiosis and cognitive impairment have been previously demonstrated [32, 33]. In the female twins, we explored gut microbiota as a potential mechanism linking diet and cognitive performance. In agreement with others, Ruminococcaceae genus was related to less decline in age-related cognition in the sample [34], and we observed novel links between MIND diet adherence and higher abundance of Ruminococcaceae *UCG-010*. Ruminococcaceae produces short-chain fatty acids (SCFA) from fibre fermentation in the gut [35]; hence, it is unsurprising that adjusting for overall dietary fibre attenuated the association. Interestingly, SCFA may protect against cognitive impairment by exerting anti-inflammatory effects, regulating neurotropic factors and reducing amyloid burden in the brain [36]. These exploratory findings suggesting beneficial effects of MIND on SCFA-producing gut bacteria linked to healthy cognitive ageing warrant further investigation.

A major strength of the present study is the twin design and the incorporation of co-twin control study to reduce confounding from genetic and shared early life environmental factors known to influence cognitive ageing. A further strength is the assessment of dietary intake using an extensively validated FFQ [19] and the inclusion of a cognitive test battery to assess individual cognitive domains that may be sensitive to diet in younger cognitively healthy populations.

This study has several limitations. The small sample of female twins as well as discordant co-twin pairs limits the power to detect causal effects of diet patterns on cognitive performance, and our findings require confirmation in larger twin studies. Furthermore, trajectories of cognitive ageing could not be modelled as cognitive tests were only performed at two time-points. Furthermore, the computer-based cognitive tests may not be as sensitive to detect changes in cognition compared to the gold-standard interview-based neuropsychological tests. Also, the twins were generally healthy with low cardiovascular burden and high level of education which could have restricted variance in both dietary exposures and cognitive test performance. While the observed effect sizes between the diet score and 10-year change in cognitive tests were small as expected, they were similar to other twin studies investigating lifestyle and cognition [37, 38]. Furthermore, we examined MIND and MED dietary patterns as they have been given most attention in the diet-dementia literature but may not necessarily represent optimal combinations of food and nutrients for healthy cognitive ageing in the sample. Given the study was conducted in female twins, the results are not generalizable to men.

In summary, adhering to MIND or MED diet was associated with better episodic and visuospatial test performance over 10 years in cognitively healthy female twins. After minimizing confounding by genetic and environmental factors, effects of diet on cognitive performance were small in the current sample and require replication in other studies. Even so, modifying diet for even small improvements in cognitive function could have important public health benefits given the increasing prevalence of AD in ageing populations. Further longitudinal studies that assess diet and changes in individual cognitive functions from midlife onwards and further examine the mediating effect of the gut microbiome will help to understand the influence of dietary patterns on trajectories of cognitive decline in ageing.

### Supplementary Information

**Additional file 1: Table S1.** Baseline cognitive performance by increasing diet score in the female twins (*n* = 509 max). **Table S2.** Mean (SE) 10-year change in cognitive performance by low and high categories of diet score (*n* =275max). **Table S3.** Associations between baseline MIND diet score and relative abundance of genus level taxa at 10-year follow-up in 141 female twins. **Table S4.** Associations between baseline MED diet score and relative abundance of genus level taxa at 10-year follow-up in 141 female twins.

## Data Availability

The data sets analysed in the current study can be made available from the corresponding author upon reasonable request.
