# Supplementary material for: Diet patterns and cognitive performance in a UK Female Twin Registry (TwinsUK)
Source: Alzheimers Res Ther. 2024 Jan 23;16:17. doi: 10.1186/s13195-024-01387-x (PMC10804649; doi:10.1186/s13195-024-01387-x)
Supplement: Supplementary file 1 — Additional file 1: Table S1. Baseline cognitive performance by increasing diet score in the female twins (n = 509 max). Table S2. Mean (SE) 10-year change in cognitive performance by low and high categories of diet score (n =275max). Table S3. Associations between baseline MIND diet score and relative abundance of genus level taxa at 10-year follow-up in 141 female twins. Table S4. Associations between baseline MED diet score and relative abundance of genus level taxa at 10-year follow-up in 141 female twins. [file 13195_2024_1387_MOESM1_ESM.docx]

**Table S1: Baseline cognitive performance by increasing diet score in the female twins (n = 509 max)**

|  | Per 1 point increase in MIND score | | | | | Per 1-point increase in MED score | | | | | | |  |
| --- | --- | --- | --- | --- | --- | --- | --- | --- | --- | --- | --- | --- | --- |
|  | **β (95% CI)** | | | | | **β (95% CI)** | | | | | | |  |
|  | **Unadjusted** |  | **Model 1** |  | **Model 2** |  | **Unadjusted** |  | **Model 1** |  | **Model 2** |  | |
| SRT  (ms) | -0.01  (-0.02, 0.00) |  | -0.01  (-0.02, 0.00) |  | -0.01*  (-0.02, 0.00) |  | -0.00  (-0.01, 0.01) |  | -0.01  (-0.02, 0.00) |  | -0.01  (-0.02, 0.00) |  | |
| SWM  (total errors) | 0.04  (-0.01, 0.08) |  | 0.02  (-0.02, 0.06) |  | 0.02  (-0.02, 0.06) |  | 0.02  (-0.02, 0.06) |  | 0.01  (-0.03, 0.04) |  | 0.00  (-0.04, 0.04) |  | |
| PAL  (total errors) | 0.01  (-0.10, 0.11) |  | -0.02  (-0.11, 0.08) |  | -0.02  (-0.12, 0.08) |  | 0.02  (-0.07, 0.11) |  | 0.00  (-0.08, 0.08) |  | 0.00  (-0.08, 0.09) |  | |
| PRM  (total correct) | 0.00  (-0.00, 0.01) |  | 0.01  (-0.00, 0.12) |  | 0.01*  (-0.00, 0.00) |  | 0.00  (-0.00, 0.01) |  | 0.00  (-0.00, 0.01) |  | 0.01  (-0.00, 0.01) |  | |
| DMS  (total correct) | -0.01  (-0.01, 0.00) |  | -0.01  (-0.01, 0.00) |  | -0.01  (-0.01, 0.00) |  | -0.01  (-0.01, 0.00) |  | -0.00  (-0.01, 0.00) |  | -0.01  (-0.01, 0.00) |  | |
| SSP  (Span) | **-0.01***  (-0.03 -0.00) |  | -0.01  (-0.02, 0.00) |  | -0.01  (-0.02, 0.00) |  | -0.01  (-0.02, 0.00) |  | -0.01  (-0.02, 0.00) |  | -0.01  (-0.02, 0.00) |  | |

Model 1: age, SES, educational attainment; Model 2: Model 1 + energy intake, BMI, physical activity, postmenopausal status, current smoking, hypertension

*P <0.05; q-value 0.35

**Table S2: Mean (SE) 10-year change in cognitive performance by low and high categories of diet score (n =275max)**

|  |  | **MIND DIET SCORE** | |  |  | **MED DIET SCORE** | | |
| --- | --- | --- | --- | --- | --- | --- | --- | --- |
|  |  | **LOW** | **HIGH** | *P-value* |  | **LOW** | **HIGH** |  |
|  | *n* | 143 | 132 |  |  | 136 | 139 | *P-value* |
| **Age-Related Cognition** | Unadjusted | 0.02 (0.09) | 0.04 (0.09) | 0.84 |  | -0.04 (0.09) | 0.09 (0.09) | 0.29 |
|  | Model 1 | 0.04 (0.08) | 0.02 (0.08) | 0.81 |  | -0.00 (0.08) | 0.06 (0.08) | 0.63 |
|  | Model 2 | 0.03 (0.08) | 0.03 (0.08) | 0.98 |  | 0.01 (0.09) | 0.05 (0.08) | 0.77 |
| **Change in SRT**  (ms) | Basic | -62.2 (4.43) | -64.7 (4.54) | 0.69 |  | -61.7 (4.52) | -65.1 (4.45) | 0.60 |
|  | Model 1 | -62.8 (4.35) | -64.1 (4.45) | 0.84 |  | -62.3 (4.44) | -64.5 (4.37) | 0.66 |
|  | Model 2 | -62.9 (4.46) | -64.0 (4.57) | 0.87 |  | -62.5 (4.46) | -64.4 (4.57) | 0.76 |
| **Change in SWM**  (total errors) | Basic | 1.09 (1.20) | 1.64 (1.25) | 0.75 |  | 2.46 (1.23) | 0.27 (1.21) | 0.21 |
|  | Model 1 | 0.84 (1.18) | 1.90 (1.22) | 0.54 |  | 2.35 (1.21) | 0.38 (1.19) | 0.25 |
|  | Model 2 | 1.12 (1.17) | 1.60 (1.22) | 0.78 |  | 2.25 (1.18) | 0.47 (1.16) | 0.29 |
| **Change in PAL**  (total errors) | Basic | 1.60 (1.23) | -1.65 (1.29) | 0.07 |  | 2.43 (1.25) | -2.30 (1.23) | 0.008** |
|  | Model 1 | 1.52 (1.23) | -1.56 (1.28) | 0.09 |  | 2.22 (1.25) | -2.09 (1.24) | 0.02* |
|  | Model 2 | 2.05 (1.25) | -2.14 (1.30) | 0.02* |  | 2.28 (1.25) | -2.15 (1.24) | 0.01* |
| **Change in PRM** (total correct) | Basic | 0.05 (0.18) | -0.27 (0.18) | 0.22 |  | -0.12 (0.18) | -0.09 (0.18) | 0.93 |
|  | Model 1 | 0.05 (0.18) | -0.27 (0.18) | 0.19 |  | -0.10 (0.18) | -0.11 (0.18) | 0.98 |
|  | Model 2 | -0.01 (0.18) | -0.21 (0.18) | 0.50 |  | -010 (0.18) | -0.11 (0.18) | 0.94 |
| **Change in DMS^2^**  (total correct) | Basic | 17.1 (0.27) | 17.6 (0.27) | 0.24 |  | 17.3 (0.27) | 17.4 (0.26) | 0.73 |
|  | Model 1 | 17.2 (0.25) | 17.5 (0.25) | 0.41 |  | 17.3 (0.25) | 17.3 (0.25) | 0.99 |
|  | Model 2 | 17.1 (0.25) | 17.6 (0.26) | 0.24 |  | 17.3 (0.25) | 17.3 (0.25) | 0.91 |
| **Change in SSP**  (Span) | Basic | -0.24 (0.08) | -0.22 (0.08) | 0.87 |  | -0.26 (0.08) | -0.20 (0.08) | 0.61 |
|  | Model 1 | -0.23 (0.08) | -0.23 (0.08) | 0.97 |  | -0.26 (0.08) | -0.20 (0.08) | 0.58 |
|  | Model 2 | -0.24 (0.08) | -0.22 (0.08) | 0.85 |  | -0.25 (0.08) | -0.21 (0.08) | 0.76 |

Basic model adjusted for baseline cognitive test score. Model 1: age, SES, educational attainment; Model 2: Model 1 + energy intake, BMI, physical activity, postmenopausal status, current smoking, hypertension. *q-value 0.21

**Table S3: Associations between baseline MIND diet score and relative abundance of genus level taxa at 10-year follow-up in 141 female twins.**

| **Taxa** | **β (95% CI)** | **q-value** |
| --- | --- | --- |
| Acidaminococcus | -0.11 (-0.28, 0.06) | 0.74 |
| Actinomyces | 0.00 (-0.01, 0.01) | 0.84 |
| Agathobacter | -0.28 (-1.68, 1.11) | 0.86 |
| Akkermansia | -1.84 (-5.44, 1.75) | 0.80 |
| Alistipes | 1.48 (-0.19, 3.16) | 0.68 |
| Allisonella | -0.01 (-0.03, 0.02) | 0.84 |
| Anaerofilum | 0.02 (0.00, 0.04) | 0.68 |
| Anaeroplasma | 0.23 (-0.02, 0.49) | 0.68 |
| Anaerostipes | -0.24 (-0.89, 0.41) | 0.83 |
| Angelakisella | 0.00 (-0.01, 0.01) | 0.95 |
| Bacteroides | 2.10 (-2.86, 7.07) | 0.81 |
| Barnesiella | 0.14 (-0.35, 0.63) | 0.84 |
| Bifidobacterium | -0.28 (-2.09, 1.52) | 0.89 |
| Bilophila | -0.05 (-0.13, 0.04) | 0.78 |
| Blautia | -1.46 (-2.80, -0.13) | 0.68 |
| Butyricicoccus | 0.02 (-0.02, 0.06) | 0.80 |
| Butyricimonas | 0.04 (-0.06, 0.15) | 0.82 |
| Butyrivibrio | 0.32 (0.02, 0.63) | 0.68 |
| CAG-352 | 0.12 (-0.05, 0.30) | 0.74 |
| CAG-56 | 0.00 (-0.07, 0.07) | 0.95 |
| Caproiciproducens | -0.07 (-0.21, 0.07) | 0.81 |
| Catenibacterium | -0.17 (-0.47, 0.14) | 0.78 |
| Christensenellaceae_R-7_group | 0.24 (-0.85, 1.34) | 0.86 |
| Clostridium_sensu_stricto | -0.18 (-0.56, 0.20) | 0.81 |
| Collinsella | -0.08 (-0.20, 0.04) | 0.74 |
| Coprobacter | 0.03 (-0.02, 0.08) | 0.74 |
| Coprococcus | -0.02 (-0.08, 0.03) | 0.81 |
| Coprococcus_2 | 0.10 (-0.46, 0.65) | 0.89 |
| Coprococcus_3 | -0.01 (-0.12, 0.10) | 0.92 |
| DTU089 | -0.06 (-0.18, 0.05) | 0.78 |
| Defluviitaleaceae_UCG-011 | 0.00 (-0.01, 0.02) | 0.83 |
| Desulfovibrio | 0.01 (-0.11, 0.14) | 0.92 |
| Dialister | -0.13 (-0.85, 0.59) | 0.89 |
| Dielma | 0.00 (-0.02, 0.03) | 0.84 |
| Dorea | -0.68 (-1.00, -0.36) | 0.01 |
| Eisenbergiella | 0.04 (-0.03, 0.10) | 0.78 |
| Enterorhabdus | 0.00 (-0.02, 0.02) | 0.95 |
| Erysipelatoclostridium | -0.06 (-0.24, 0.12) | 0.83 |
| Erysipelotrichaceae_UCG-003 | -0.02 (-0.46, 0.43) | 0.96 |
| Escherichia/Shigella | -0.10 (-3.18, 2.98) | 0.96 |
| Faecalibacterium | -0.13 (-1.39, 1.14) | 0.92 |
| Faecalitalea | 0.00 (-0.03, 0.04) | 0.90 |
| Family_XIII_AD3011_group | 0.05 (-0.03, 0.13) | 0.74 |
| Family_XIII_UCG-001 | 0.00 (-0.02, 0.02) | 0.89 |
| Flavonifractor | 0.00 (-0.04, 0.05) | 0.95 |
| Fournierella | 0.03 (0.00, 0.05) | 0.68 |
| Fusicatenibacter | 0.03 (-0.47, 0.53) | 0.95 |
| GCA-900066225 | 0.01 (-0.01, 0.03) | 0.81 |
| GCA-900066575 | 0.00 (-0.01, 0.02) | 0.84 |
| Gordonibacter | 0.00 (-0.01, 0.02) | 0.84 |
| Haemophilus | 0.05 (-0.20, 0.31) | 0.86 |
| Holdemanella | -0.18 (-0.55, 0.19) | 0.81 |
| Holdemania | 0.00 (-0.01, 0.01) | 0.84 |
| Howardella | 0.01 (0.00, 0.02) | 0.74 |
| Hungatella | 0.05 (-0.03, 0.14) | 0.74 |
| Hydrogenoanaerobacterium | 0.00 (0.00, 0.01) | 0.83 |
| Intestinibacter | 0.06 (-0.35, 0.47) | 0.90 |
| Intestinimonas | -0.01 (-0.04, 0.03) | 0.84 |
| Klebsiella | -0.17 (-0.77, 0.43) | 0.84 |
| Lachnoclostridium | -0.20 (-0.52, 0.12) | 0.74 |
| Lachnospira | 0.02 (-0.35, 0.39) | 0.95 |
| Lachnospiraceae_AC2044_group | 0.06 (0.02, 0.10) | 0.27 |
| Lachnospiraceae_FCS020_group | -0.02 (-0.04, 0.01) | 0.74 |
| Lachnospiraceae_ND3007_group | -0.12 (-0.25, 0.00) | 0.68 |
| Lachnospiraceae_NK4A136_group | 0.04 (-0.36, 0.44) | 0.92 |
| Lachnospiraceae_NK4B4_group | 0.00 (-0.01, 0.02) | 0.89 |
| Lachnospiraceae_UCG-001 | -0.01 (-0.11, 0.08) | 0.89 |
| Lachnospiraceae_UCG-003 | 0.03 (0.00, 0.05) | 0.68 |
| Lachnospiraceae_UCG-004 | 0.00 (-0.05, 0.05) | 0.94 |
| Lachnospiraceae_UCG-008 | 0.00 (-0.01, 0.01) | 0.83 |
| Lachnospiraceae_UCG-010 | 0.01 (-0.04, 0.06) | 0.86 |
| Lactobacillus | -0.12 (-0.26, 0.01) | 0.68 |
| Marvinbryantia | -0.03 (-0.11, 0.04) | 0.81 |
| Merdibacter | -0.01 (-0.03, 0.00) | 0.74 |
| Methanobrevibacter | 0.30 (-0.46, 1.06) | 0.82 |
| Negativibacillus | -0.02 (-0.06, 0.01) | 0.74 |
| Odoribacter | 0.05 (-0.01, 0.10) | 0.68 |
| Oscillibacter | 0.02 (-0.05, 0.09) | 0.84 |
| Oscillospira | -0.03 (-0.05, 0.00) | 0.68 |
| Oxalobacter | 0.02 (0.01, 0.03) | 0.17 |
| Parabacteroides | 0.26 (-0.53, 1.04) | 0.84 |
| Paraprevotella | -0.31 (-0.76, 0.14) | 0.74 |
| Parasutterella | -0.05 (-0.19, 0.08) | 0.82 |
| Peptococcus | -0.02 (-0.04, 0.01) | 0.74 |
| Phascolarctobacterium | -0.08 (-0.26, 0.10) | 0.81 |
| Prevotella | 0.01 (-0.01, 0.03) | 0.82 |
| Prevotella_7 | 0.17 (-0.28, 0.62) | 0.83 |
| Prevotella_9 | -0.73 (-2.91, 1.45) | 0.83 |
| Rikenellaceae_RC9_gut_group | 0.21 (-0.62, 1.03) | 0.84 |
| Romboutsia | -0.06 (-0.23, 0.12) | 0.84 |
| Roseburia | -0.54 (-1.25, 0.17) | 0.74 |
| Ruminiclostridium_5 | -0.12 (-0.26, 0.01) | 0.68 |
| Ruminiclostridium_6 | 0.10 (-0.13, 0.34) | 0.81 |
| Ruminiclostridium_9 | -0.03 (-0.08, 0.03) | 0.81 |
| Ruminococcaceae_NK4A214_group | 0.32 (0.08, 0.56) | 0.30 |
| Ruminococcaceae_UCG-002 | 0.86 (-0.30, 2.03) | 0.74 |
| Ruminococcaceae_UCG-003 | 0.06 (-0.04, 0.16) | 0.74 |
| Ruminococcaceae_UCG-004 | -0.01 (-0.02, 0.01) | 0.83 |
| Ruminococcaceae_UCG-005 | 0.09 (-0.43, 0.60) | 0.89 |
| Ruminococcaceae_UCG-009 | 0.01 (0.00, 0.01) | 0.68 |
| Ruminococcaceae_UCG-010 | 0.39 (0.17, 0.62) | 0.05 |
| Ruminococcaceae_UCG-011 | 0.00 (-0.01, 0.01) | 0.84 |
| Ruminococcaceae_UCG-013 | 0.12 (-0.05, 0.28) | 0.74 |
| Ruminococcaceae_UCG-014 | 0.28 (-0.85, 1.41) | 0.84 |
| Ruminococcus | -0.14 (-0.82, 0.54) | 0.86 |
| Ruminococcus_2 | -0.12 (-1.16, 0.92) | 0.92 |
| Sellimonas | 0.00 (-0.01, 0.01) | 0.96 |
| Senegalimassilia | -0.02 (-0.05, 0.02) | 0.81 |
| Shuttleworthia | 0.06 (-0.07, 0.19) | 0.81 |
| Slackia | 0.00 (-0.01, 0.01) | 0.84 |
| Streptococcus | -0.08 (-0.39, 0.23) | 0.84 |
| Subdoligranulum | -0.32 (-1.22, 0.58) | 0.83 |
| Sutterella | 0.11 (-0.05, 0.27) | 0.74 |
| Terrisporobacter | 0.04 (-0.09, 0.16) | 0.84 |
| Turicibacter | -0.03 (-0.09, 0.03) | 0.78 |
| Tyzzerella | -0.04 (-0.12, 0.05) | 0.81 |
| Tyzzerella_3 | -0.04 (-0.08, 0.01) | 0.74 |
| Tyzzerella_4 | -0.01 (-0.05, 0.04) | 0.89 |
| UBA1819 | 0.05 (-0.10, 0.21) | 0.83 |
| UC5-1-2E3 | -0.01 (-0.03, 0.01) | 0.83 |
| Veillonella | 0.10 (-0.08, 0.27) | 0.78 |
| Victivallis | 0.01 (0.00, 0.01) | 0.78 |
| unclassified Barnesiellaceae | 0.05 (0.00, 0.10) | 0.68 |
| unclassified Christensenellaceae | 0.21 (-0.07, 0.50) | 0.74 |
| unclassified Clostridiales_vadinBB60_group | 0.21 (-0.07, 0.49) | 0.74 |
| unclassified Coriobacteriales_Incertae_Sedis | -0.07 (-0.15, 0.01) | 0.68 |
| unclassified Desulfovibrionaceae | 0.00 (-0.01, 0.02) | 0.84 |
| unclassified Eggerthellaceae | -0.01 (-0.03, 0.01) | 0.81 |
| unclassified Erysipelotrichaceae | 0.02 (-0.01, 0.05) | 0.78 |
| unclassified Family_XIII | 0.01 (-0.01, 0.02) | 0.84 |
| unclassified Lachnospiraceae | -0.01 (-0.66, 0.64) | 0.97 |
| unclassified Muribaculaceae | 0.05 (-0.35, 0.46) | 0.90 |
| unclassified NA | 0.56 (-0.71, 1.83) | 0.81 |
| unclassified Peptococcaceae | 0.01 (-0.01, 0.03) | 0.78 |
| unclassified Ruminococcaceae | -0.36 (-1.61, 0.89) | 0.84 |

Values are beta coefficients (95% CI) representing the difference in relative abundance of taxa between low and high diet scores. MIND diet scores were 0-7.5 for the low adherence group and 8-15 for the high adherence group. Model adjusted for age, SES, educational attainment, energy intake, BMI, physical activity, postmenopausal status, current smoking, hypertension. Q-value= false discovery rate adjusted *P*-value.

**Table S4: Associations between baseline MED diet score and relative abundance of genus level taxa at 10-year follow-up in 141 female twins.**

| **Taxa** | **β (95% CI)** | **Q-value** |
| --- | --- | --- |
| Acidaminococcus | -0.05 (-0.24, 0.15) | 0.89 |
| Actinomyces | 0.00 (-0.01, 0.01) | 0.99 |
| Agathobacter | -0.24 (-1.52, 1.04) | 0.94 |
| Akkermansia | 1.57 (-0.74, 3.89) | 0.81 |
| Alistipes | 1.28 (-0.37, 2.94) | 0.81 |
| Allisonella | 0.01 (-0.01, 0.02) | 0.87 |
| Anaerofilum | 0.00 (-0.02, 0.02) | 0.99 |
| Anaeroplasma | -0.07 (-0.49, 0.35) | 0.95 |
| Anaerostipes | -0.18 (-0.64, 0.29) | 0.84 |
| Angelakisella | -0.01 (-0.02, 0.00) | 0.81 |
| Bacteroides | 3.04 (-1.46, 7.54) | 0.81 |
| Barnesiella | 0.23 (-0.27, 0.73) | 0.82 |
| Bifidobacterium | 0.02 (-1.51, 1.54) | 0.99 |
| Bilophila | -0.02 (-0.09, 0.05) | 0.89 |
| Blautia | -0.85 (-2.49, 0.79) | 0.81 |
| Butyricicoccus | 0.00 (-0.04, 0.04) | 0.99 |
| Butyricimonas | -0.01 (-0.13, 0.10) | 0.97 |
| Butyrivibrio | 0.11 (-0.14, 0.37) | 0.82 |
| CAG-352 | 0.08 (-0.09, 0.25) | 0.82 |
| CAG-56 | -0.03 (-0.09, 0.03) | 0.81 |
| Caproiciproducens | 0.06 (-0.06, 0.19) | 0.81 |
| Catenibacterium | -0.06 (-0.39, 0.27) | 0.94 |
| Christensenellaceae_R-7_group | 0.02 (-1.23, 1.27) | 0.99 |
| Clostridium_sensu_stricto | -0.04 (-0.47, 0.39) | 0.98 |
| Collinsella | -0.02 (-0.10, 0.05) | 0.87 |
| Coprobacter | 0.02 (-0.02, 0.06) | 0.82 |
| Coprococcus | -0.05 (-0.10, 0.01) | 0.81 |
| Coprococcus_2 | 0.32 (-0.24, 0.88) | 0.81 |
| Coprococcus_3 | -0.08 (-0.21, 0.05) | 0.81 |
| DTU089 | -0.07 (-0.24, 0.10) | 0.82 |
| Defluviitaleaceae_UCG-011 | 0.00 (-0.01, 0.01) | 0.99 |
| Desulfovibrio | -0.07 (-0.18, 0.04) | 0.81 |
| Dialister | -0.29 (-1.18, 0.61) | 0.87 |
| Dielma | 0.02 (-0.03, 0.06) | 0.84 |
| Dorea | -0.48 (-0.80, -0.16) | 0.56 |
| Eisenbergiella | 0.04 (-0.03, 0.10) | 0.81 |
| Enterorhabdus | -0.01 (-0.03, 0.01) | 0.87 |
| Erysipelatoclostridium | -0.02 (-0.17, 0.13) | 0.97 |
| Erysipelotrichaceae_UCG-003 | 0.21 (-0.31, 0.72) | 0.82 |
| Escherichia/Shigella | -1.75 (-4.59, 1.09) | 0.81 |
| Faecalibacterium | -0.50 (-1.74, 0.73) | 0.82 |
| Faecalitalea | -0.02 (-0.06, 0.02) | 0.81 |
| Family_XIII_AD3011_group | 0.05 (-0.05, 0.14) | 0.81 |
| Family_XIII_UCG-001 | -0.01 (-0.03, 0.01) | 0.87 |
| Flavonifractor | 0.02 (-0.02, 0.07) | 0.81 |
| Fournierella | 0.02 (-0.01, 0.05) | 0.81 |
| Fusicatenibacter | -0.22 (-0.75, 0.31) | 0.82 |
| GCA-900066225 | -0.01 (-0.03, 0.02) | 0.87 |
| GCA-900066575 | 0.00 (-0.01, 0.01) | 0.99 |
| Gordonibacter | 0.01 (0.00, 0.03) | 0.81 |
| Haemophilus | -0.02 (-0.21, 0.17) | 0.97 |
| Holdemanella | -0.27 (-0.61, 0.06) | 0.81 |
| Holdemania | 0.01 (-0.01, 0.02) | 0.81 |
| Howardella | 0.00 (-0.01, 0.02) | 0.89 |
| Hungatella | 0.05 (-0.04, 0.14) | 0.81 |
| Hydrogenoanaerobacterium | 0.00 (0.00, 0.01) | 0.81 |
| Intestinibacter | -0.02 (-0.36, 0.31) | 0.99 |
| Intestinimonas | 0.01 (-0.02, 0.05) | 0.87 |
| Klebsiella | 0.47 (-0.27, 1.21) | 0.81 |
| Lachnoclostridium | 0.05 (-0.24, 0.34) | 0.94 |
| Lachnospira | 0.10 (-0.23, 0.42) | 0.87 |
| Lachnospiraceae_AC2044_group | 0.01 (-0.04, 0.06) | 0.94 |
| Lachnospiraceae_FCS020_group | -0.02 (-0.05, 0.00) | 0.81 |
| Lachnospiraceae_ND3007_group | -0.13 (-0.25, -0.01) | 0.81 |
| Lachnospiraceae_NK4A136_group | -0.19 (-0.60, 0.22) | 0.82 |
| Lachnospiraceae_NK4B4_group | 0.00 (-0.02, 0.01) | 0.97 |
| Lachnospiraceae_UCG-001 | 0.00 (-0.10, 0.09) | 0.99 |
| Lachnospiraceae_UCG-003 | -0.01 (-0.05, 0.02) | 0.87 |
| Lachnospiraceae_UCG-004 | 0.00 (-0.04, 0.05) | 0.97 |
| Lachnospiraceae_UCG-008 | 0.00 (-0.01, 0.01) | 0.97 |
| Lachnospiraceae_UCG-010 | 0.04 (-0.01, 0.10) | 0.81 |
| Lactobacillus | -0.06 (-0.16, 0.05) | 0.81 |
| Marvinbryantia | -0.05 (-0.13, 0.03) | 0.81 |
| Merdibacter | 0.01 (-0.01, 0.03) | 0.84 |
| Methanobrevibacter | 0.19 (-0.53, 0.91) | 0.89 |
| Negativibacillus | -0.02 (-0.06, 0.01) | 0.81 |
| Odoribacter | 0.04 (-0.01, 0.10) | 0.81 |
| Oscillibacter | 0.02 (-0.05, 0.08) | 0.89 |
| Oscillospira | -0.01 (-0.03, 0.01) | 0.82 |
| Oxalobacter | 0.01 (0.00, 0.02) | 0.81 |
| Parabacteroides | 0.26 (-0.51, 1.03) | 0.87 |
| Paraprevotella | -0.30 (-0.76, 0.15) | 0.81 |
| Parasutterella | -0.09 (-0.20, 0.02) | 0.81 |
| Peptococcus | 0.01 (-0.03, 0.05) | 0.89 |
| Phascolarctobacterium | -0.08 (-0.21, 0.05) | 0.81 |
| Prevotella | -0.02 (-0.05, 0.01) | 0.81 |
| Prevotella_7 | 0.08 (-0.26, 0.42) | 0.89 |
| Prevotella_9 | 0.65 (-0.93, 2.22) | 0.82 |
| Rikenellaceae_RC9_gut_group | -0.03 (-0.75, 0.69) | 0.99 |
| Romboutsia | 0.02 (-0.16, 0.20) | 0.97 |
| Roseburia | -0.28 (-0.97, 0.42) | 0.83 |
| Ruminiclostridium_5 | -0.04 (-0.20, 0.12) | 0.89 |
| Ruminiclostridium_6 | -0.07 (-0.31, 0.17) | 0.89 |
| Ruminiclostridium_9 | -0.05 (-0.10, 0.01) | 0.81 |
| Ruminococcaceae_NK4A214_group | 0.01 (-0.31, 0.33) | 0.99 |
| Ruminococcaceae_UCG-002 | -0.28 (-1.41, 0.85) | 0.89 |
| Ruminococcaceae_UCG-003 | -0.01 (-0.07, 0.06) | 0.99 |
| Ruminococcaceae_UCG-004 | 0.00 (-0.01, 0.01) | 0.92 |
| Ruminococcaceae_UCG-005 | -0.07 (-0.67, 0.54) | 0.97 |
| Ruminococcaceae_UCG-009 | 0.00 (-0.01, 0.00) | 0.89 |
| Ruminococcaceae_UCG-010 | 0.16 (-0.08, 0.39) | 0.81 |
| Ruminococcaceae_UCG-011 | 0.00 (-0.01, 0.00) | 0.81 |
| Ruminococcaceae_UCG-013 | 0.02 (-0.11, 0.15) | 0.97 |
| Ruminococcaceae_UCG-014 | -1.26 (-2.33, -0.20) | 0.81 |
| Ruminococcus | 0.02 (-0.62, 0.67) | 0.99 |
| Ruminococcus_2 | -0.35 (-1.48, 0.78) | 0.87 |
| Sellimonas | 0.01 (0.00, 0.01) | 0.81 |
| Senegalimassilia | -0.03 (-0.08, 0.02) | 0.81 |
| Shuttleworthia | 0.08 (-0.07, 0.23) | 0.81 |
| Slackia | -0.01 (-0.02, 0.00) | 0.81 |
| Streptococcus | 0.04 (-0.29, 0.37) | 0.97 |
| Subdoligranulum | -0.27 (-1.11, 0.58) | 0.87 |
| Sutterella | 0.06 (-0.11, 0.22) | 0.87 |
| Terrisporobacter | 0.01 (-0.10, 0.12) | 0.98 |
| Turicibacter | -0.07 (-0.14, 0.01) | 0.81 |
| Tyzzerella | 0.06 (-0.02, 0.14) | 0.81 |
| Tyzzerella_3 | -0.02 (-0.06, 0.01) | 0.81 |
| Tyzzerella_4 | -0.02 (-0.07, 0.02) | 0.81 |
| UBA1819 | 0.13 (0.00, 0.27) | 0.81 |
| UC5-1-2E3 | 0.00 (-0.03, 0.02) | 0.97 |
| Veillonella | 0.13 (-0.17, 0.44) | 0.82 |
| Victivallis | 0.00 (-0.01, 0.01) | 0.89 |
| unclassified Barnesiellaceae | 0.02 (-0.01, 0.05) | 0.81 |
| unclassified Christensenellaceae | -0.08 (-0.35, 0.19) | 0.87 |
| unclassified Clostridiales_vadinBB60_group | 0.19 (-0.20, 0.59) | 0.81 |
| unclassified Coriobacteriales_Incertae_Sedis | -0.07 (-0.14, 0.01) | 0.81 |
| unclassified Desulfovibrionaceae | -0.01 (-0.03, 0.01) | 0.81 |
| unclassified Eggerthellaceae | -0.01 (-0.03, 0.01) | 0.81 |
| unclassified Erysipelotrichaceae | 0.02 (-0.01, 0.05) | 0.81 |
| unclassified Family_XIII | 0.00 (-0.02, 0.02) | 0.99 |
| unclassified Lachnospiraceae | -0.06 (-0.74, 0.63) | 0.98 |
| unclassified Muribaculaceae | 0.00 (-0.30, 0.31) | 0.99 |
| unclassified NA | 0.22 (-0.87, 1.31) | 0.92 |
| unclassified Peptococcaceae | -0.01 (-0.03, 0.01) | 0.82 |
| unclassified Ruminococcaceae | -0.84 (-2.15, 0.47) | 0.81 |

Values are beta coefficients (95% CI) representing the difference in relative abundance of taxa between low and high diet scores. MED diet scores were 0-4 for the low adherence group and 5-9 for the high adherence group. Model adjusted for age, SES, educational attainment, energy intake, BMI, physical activity, postmenopausal status, current smoking, hypertension. Q-value= false discovery rate adjusted *P*-value.
